# Radiomics Analysis of Fat-Saturated T2-Weighted MRI Sequences for the Prediction of Prognosis in Soft Tissue Sarcoma of the Extremities and Trunk Treated With Neoadjuvant Radiotherapy

**DOI:** 10.3389/fonc.2021.710649

**Published:** 2021-09-17

**Authors:** Silin Chen, Ning Li, Yuan Tang, Bo Chen, Hui Fang, Shunan Qi, Ninging Lu, Yong Yang, Yongwen Song, Yueping Liu, Shulian Wang, Ye-xiong Li, Jing Jin

**Affiliations:** ^1^Department of Radiation Oncology, National Cancer Center/National Clinical Research Center for Cancer/Cancer Hospital, Chinese Academy of Medical Sciences and Peking Union Medical College, Beijing, China; ^2^Department of Radiation Oncology, National Cancer Center/National Clinical Research Center for Cancer/Cancer Hospital & Shenzhen Hospital, Chinese Academy of Medical Sciences and Peking Union Medical College, Shenzhen, China

**Keywords:** sarcoma, neoadjuvant therapy, magnetic resonance imaging, radiomic, prognosis

## Abstract

**Purpose:**

To create a prognostic prediction radiomics model for soft tissue sarcoma (STS) of the extremities and trunk treated with neoadjuvant radiotherapy.

**Methods:**

This study included 62 patients with STS of the extremities and trunk who underwent magnetic resonance imaging (MRI) before neoadjuvant radiotherapy. After tumour segmentation and preprocessing, 851 radiomics features were extracted. The radiomics score was constructed according to the least absolute shrinkage and selection operator (LASSO) method. Survival analysis (disease-free survival; DFS) was performed using the log-rank test and Cox’s proportional hazards regression model. The nomogram model was established based on the log-rank test and Cox regression model. Harrell’s concordance index (C-index), calibration curve and receiver operating characteristic (ROC) curve analysis were used to evaluate the prognostic factors. The clinical utility of the model was assessed by decision curve analysis (DCA).

**Results:**

The univariate survival analysis showed that tumour location (p = 0.032), clinical stage (p = 0.022), tumour size (p = 0.005) and the radiomics score were correlated with DFS (p < 0.05). The multivariate analysis showed that tumour location, tumour size, and the radiomics score were independent prognostic factors for DFS (p < 0.05). The combined clinical-radiomics model based on the multivariate analysis showed the best predictive ability for DFS (C-index: 0.781; Area Under Curve: 0.791). DCA revealed that the use of the radiomics score-based nomogram was associated with better benefit gains relative to the prediction of 2-year DFS events than other models in the threshold probability range between 0.12 and 0.38.

**Conclusion:**

The radiomics score from pretreatment MRI is an independent prognostic factor for DFS in patients with STS of the extremities and trunk. The radiomics score-based nomogram could improve prognostic stratification ability and thus contribute to individualized therapy for STS patients.

## Introduction

Soft tissue sarcoma (STS) is an uncommon malignant tumour that represents less than 1% of all newly diagnosed malignant tumours (1). The cornerstone of the management of STS patients is surgery. Tumours with close or positive margin resection have unacceptably high rates of local recurrence (2, 3). For high-risk patients with STS of the extremities and trunk, the recommended treatment is surgery combined with radiation therapy (RT). Based on the pros and cons of neoadjuvant *versus* adjuvant RT, the panel has expressed a general preference for neoadjuvant RT, which could reduce late toxicities (fibrosis, oedema, and joint stiffness), with a lower total dose of RT and a smaller treatment field size (4–6).

At present, clinical staging systems such as the TNM staging and grading systems are the most widely used prognostic markers in STS but are not efficient (7–9). Previous studies have demonstrated the prognostic value of magnetic resonance imaging (MRI) in STS (10, 11), and radiomics, a high-dimensional technology, can be used to further analyse tumour features beyond known parameters. Radiomics features (such as intensity, texture or wavelet) can offer information about the tumour microenvironment, tumour grade and long-term prognosis (12–16). There are limited studies concerning the prediction of the prognosis of neoadjuvant radiotherapy in STS.

In this study, we evaluated a radiomics model derived from MRI for the prediction of prognosis in STS of the extremities and trunk treated with neoadjuvant radiotherapy. The net benefit of the nomogram model regarding the clinical decision analysis and patient risk stratification was analysed.

## Materials and Methods

### Patients

The dataset in this study included 62 STS patients: 20 were treated with neoadjuvant radiotherapy at our institution between January 2015 and December 2019 (n=20), and 42 underwent imaging from The Cancer Imaging Archive (TCIA; NCI). between November 2004 and November 2011 (17, 18). The primary inclusion criteria for the participants were treated with neoadjuvant radiotherapy and surgery, tumour located in the extremity or trunk, and pretreatment MR images (fat-saturated T2-weighted (T2FS) sequences) available. The exclusion criteria were previous chemotherapy and metastatic and/or recurrent STSs. Baseline clinical and epidemiological characteristics, including age, sex, clinical T stage, clinical N stage, histological type, and histological grade, were obtained from medical records. Tumour stage was evaluated according to the 7th edition of the AJCC staging system (19). According to the French Federation of Cancer Centers Sarcoma Group grading system, for example, the high grade was defined as grade III (**Table 1**) (20).

**Table 1:** Patient characteristics.

Characteristics	Patients (n = 62)
**Gender**	
Male	32 (51.6)
Female	30 (48.4)
**Age at diagnosis (y), mean ± SD**	50.9 ± 19.1
**Histotype**	
Undifferentiated sarcoma	14 (22.6)
liposarcoma	15 (24.2)
Synovial sarcoma	5 (8.1)
Leiomyosarcoma	10 (16.1)
Fibrosarcoma	4 (6.5)
Other^a^	14 (22.6)
**Grade**	
Low	7 (11.3)
Intermediate	16 (25.8)
High	32 (51.6)
Unkown	7 (11.3)
**Location**	
Trunk	9 (14.5)
Extremities	53 (85.5)
**MRI T stage**	
cT1	7 (11.3)
cT2	55 (88.7)
**MRI N stage**	
cN0	62 (100.0)
**Depth**	
Superficial	16 (25.8)
Deep	46 (74.2)
**Clinical stage**	
I	7 (11.3)
II	27 (43.5)
III	28 (45.2)
**Treatment**	
Radiotherapy + Surgery	49 (79.0)
Radiotherapy + Surgery + Chemotherapy	13 (21.0)

Data are reported as No. (%).

^a^Including epithelioid sarcoma, myxofibrosarcoma, extraskeletal high grade osteogenic sarcoma, etc.

### Image Acquisition and Definition of the Region of Interest (ROI)

MR scans at the TCIA and our institution were performed with 1.5 T/3.0 T MR systems. Forty-two patients had their images acquired at the TCIA. The median in-plane resolution was 0.63 × 0.63 mm^2^ (range: 0.23–1.64 mm), the median acquisition matrix (pixels) was 512×512, and the median slice thickness was 5.0 mm (range: 3.0–8.0 mm) for T2FS sequences from the TCIA database (17). Twenty patients underwent MRI on a GE Discovery MR (750 W 3.0 T) at our institution. The acquisition matrix (pixels) was 512×512, and the median slice thickness was 5.0 mm (range: 3.0–5.0 mm). The repetition time/echo time (TR/TE) ranged from 7845–13405/92–72 msec for T2FS sequences. Tumour segmentation was conducted manually using 3D Slicer software (Slicer, version 4.10.2) (21). The ROI was drawn over the primary tumour excluding areas of peritumoural oedema in the T2FS images. To ensure accuracy and precision, the ROI was manually delineated slice by slice on the axial images. The delineation process was performed by two different radiation oncology residents, in case of the two residents do not agree on the delineation, they will reach an agreement through consultation under the guidance of senior radiation oncology professors.

### Image Preprocessing and Radiomics Feature Extraction

The open-source software 3D Slicer (version 4.10.2) was used for image segmentation. Radiomics features were extracted *via* pyradiomics (version 3.0) implementation in 3D Slicer. Radiomics features included first-order statistics (first-order), shape-based (3D) features, shape-based (2D) features, grey-level cooccurrence matrix (GLCM), grey-level run length matrix (GLRLM), grey-level size zone matrix (GLSZM), neighbouring grey tone difference matrix (NGTDM) and grey-level dependence matrix (GLDM) (17). Wavelet decomposition filtering was performed following image reconstruction. By applying different weights to the bandpass and sub-bands, the ROI was compared to low- and high-frequency sub-bands in the wavelet domain. The fixed bin width for image discretization was 25. The same voxel size (1 × 1 × 1 mm) was used for all model calculations.

### Radiomics Feature Selection and Radiomics Score Construction

To improve feature repeatability and reproducibility, the differences between the features generated by two radiation oncology residents were assessed with the intraclass correlation coefficient (ICC). An ICC value between 0.90 and 1.00 was considered reliable. To pool radiomics features extracted from the TCIA database and our institution relying on different MRI protocols, the intensities of all radiomics features were normalized by the ComBat compensation method and z score transformation (22–24). Feature selection was performed using least absolute shrinkage and selection operator (LASSO) regression in the cohort. As a compression estimation method, the LASSO method shrinks all regression coefficients towards zero and changes the coefficients of irrelevant features to zero (25). Through feature reduction and selection, the LASSO method was used to build a more refined model. The standardized constraint parameter was set to 0.002175, and 42 nonzero coefficients were selected by the LASSO method. LASSO Cox regression model analysis was performed using the “glmnet” package of R software. The radiomics score was constructed based on the LASSO regression results (26).

### Statistical Analysis

Statistical analyses were conducted using MedCalc software (19.07) and R statistical software (R version 3.6.3). The optimal cut-off value of the radiomics score and tumour size was determined according to the highest χ^2^ value defined by the log-rank test and Kaplan-Meier survival analysis using X-Tile (Rimm Laboratory, Yale University, version 3.6.1) (27). The primary outcome was disease-free survival (DFS). Survival was calculated using Kaplan–Meier survival curve analysis (log-rank test). Univariate and multivariate hazard ratios were analysed for DFS using univariate and multivariate Cox proportional hazards regression models (survival package). The nomogram was generated with the “rms” package. The nomogram model was evaluated by Harrell’s concordance index (C-index), receiver operating characteristic (ROC) curve analysis (timeROC package) and calibration curve. To evaluate the unbiased performance of the model, the model was retested for internal validation using bootstrapping (n=1000) (Boot package). To evaluate the clinical utility of the combined model, decision curve analysis (DCA) was performed according to the method of Vickers et al. (28, 29). DCA explores the clinical benefit of different models by calculating the net benefit of each decision strategy at each threshold probability (28). DFS was measured from the time of the initial imaging diagnosis until a DFS event (including death, local recurrence or metastasis) or censoring.

## Results

### Patient Characteristics

The cohort included 62 patients [30 females (48.4%), mean age: 50.9 ± 19.1 years]. The most frequent histological subtypes were liposarcoma (24.2%) and undifferentiated sarcoma (22.6%). Most tumours were deep seated (74.2%) and located in the extremity (85.5%). A total of 51.6% of patients had high-grade STS, and 25.8% had intermediate-grade STS. The median maximum tumour diameter was 9.3 cm (range 2.6-24.6), forty patients (64.5%) had maximum tumour diameter smaller than 11 cm. Forty-nine patients (79.0%) received “radiotherapy + surgery”, and thirteen patients (21.0%) received “radiotherapy + surgery + chemotherapy” (**Table 1**). The detail of two the cohorts was showed in **Supplementary Table 2**.

### Extraction of the Radiomics Features and Development of the Radiomics Score

**Figure 1:**
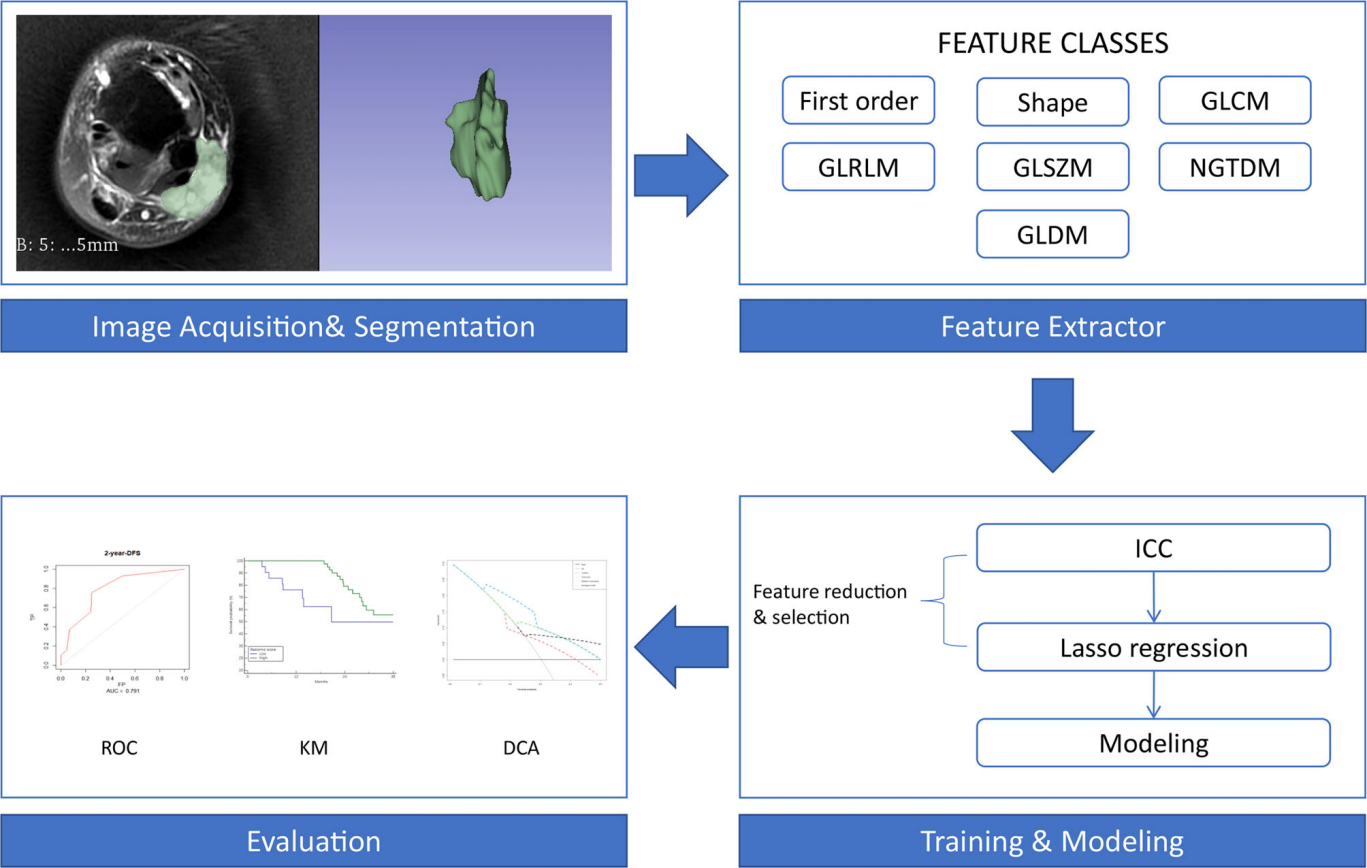
Workflow of the radiomics analysis. First-order, first-order statistics; Shape, shape-based (3D) features and shape-based (2D) features; GLCM, grey-level cooccurrence matrix; GLRLM, grey-level run length matrix; GLSZM, grey-level size zone matrix; NGTDM, neighbouring grey tone difference matrix; GLDM, grey-level dependence matrix; ICC, intraclass correlation coefficient; ROC, receiver operating characteristic curve; KM, Kaplan-Meier survival curve; DCA, decision curve analysis.

In total, 851 radiomics features were extracted from the T2FS images. After eliminating the features with low reproducibility, 777 remained. **Supplementary Figure 1** shows the data obtained following dimensionality reduction according to the LASSO method. The 42 most valuable variables remained, and their individual LASSO coefficients are shown in **Supplementary Table 1**. The formula used to calculate the radiomics score is presented in the supplemental file. The optimal cut-off value of the radiomics score determined by X-tile software was 53. Therefore, patients were divided into those with high (> 53) and low (≤ 53) radiomics scores. The workflow of the analysis is presented in **Figure 1**.

### Patient Risk Stratification

**Figure 2:**
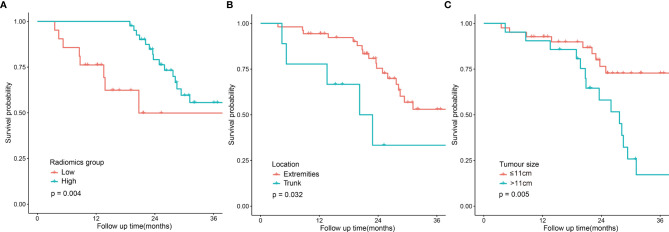
| Disease-free survival curves according to patient risk stratification. Survival curves stratified by the radiomics score (high *vs* low) **(A)**, tumour location (trunk *vs* extremities) **(B)** and tumour size (≤11 cm *vs* <11 cm) **(C)**.

The univariate analysis was performed on all the variables (**Table 2**). The median follow-up time was 25.3 months (range 4.4-70.7). The 2-year DFS for the entire cohort was 69.6% (95% CI:56.9-82.3). DFS was significantly different in terms of tumour location (p=0.032, **Figure 2B**), clinical stage (p=0.022), tumour size (p=0.005, **Figure 2C**) and the radiomics score (p=0.004). Comparison of the survival curves according to the radiomics score (high *vs* low) indicated that this stratification achieved prognostic separation for patients. Compared to the high radiomics score group, the low radiomics score group had significantly poorer DFS (2-year DFS [95% CI] for the low group [n = 21] *versus* for the high group [n = 41]: 49.9 [28.2–83.8] *versus* 79.1 [67.1–93.2]; p=0.004, **Figure 2A**). The multivariate analysis demonstrated that tumour size, tumour location and the radiomics score were independent predictors for DFS (**Table 2**).

**Table 2:** Univariate and multivariate analyses of predictors for DFS.

Variables	Univariate analyses			Multivariate analyses		
	HR	95%CI	*p*	HR	95%CI	*p*
**Gender**						
Male	1	0.238-1.323	0.187	\	\	\
Female	0.562			\	\	\
**Grade**						
Low	1	0.552-31.251	0.167	\	\	\
Intermediate-High	4.150			\	\	\
**Location**						
Extremities	1	1.100-8.232	0.032	1	1.039-11.735	0.043
Trunk	3.010			3.491		
**MRI T stage**						
cT1	1	0.108-5656.874	0.247	\	\	\
cT2	24.746			\	\	\
**Depth**						
Superficial	1	0.415-3.016	0.824	\	\	\
Deep	1.119			\	\	\
**Clinical stage**						
I-II	1	1.163-7.067	0.022	1	0.878-5.815	0.091
III	2.867			2.260		
**Tumor size**						
≤11	1	1.455-7.824	0.005	1	1.405-9.065	0.007
>11	3.374			3.569		
**Radiomics score**						
High	1	1.570-10.101	0.004	1	1.300-11.236	0.015
Low	3.984			3.817		

### Clinical Radiomics Nomogram for DFS

**Figure 3:**
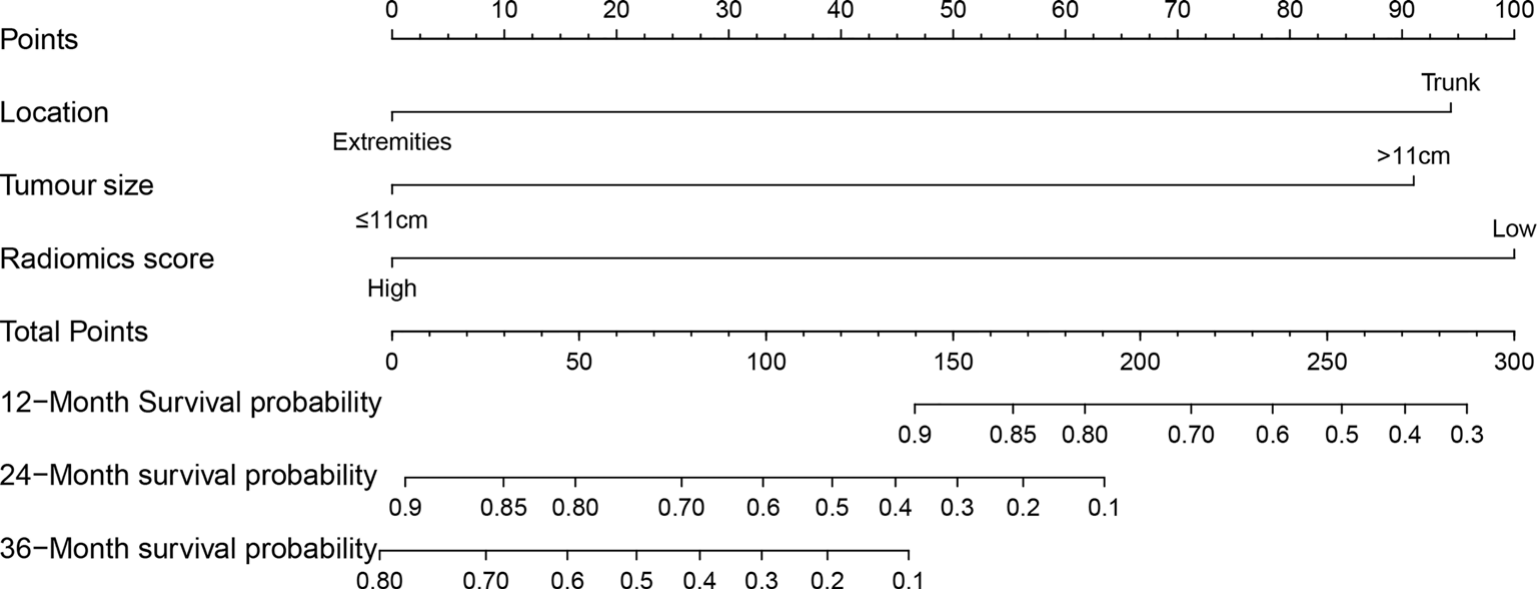
The clinical-radiomics nomogram: The radiomics score-based nomogram for the prediction of DFS was developed in the cohort, and the radiomics score, tumour location and tumour size were incorporated.

**Figure 4:**
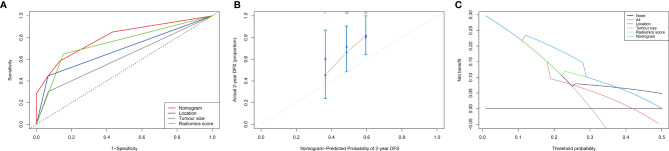
Evaluation of the radiomics score-based nomogram. **(A)** The receiver operating characteristic (ROC) curve; **(B)** Calibration curve for the radiomics nomogram in the cohort. The x-axis shows the predicted probability of a DFS event. The y-axis shows the actual DFS outcome; **(C)** Decision curve analysis (DCA) of the radiomics nomogram. The threshold probability was calculated for a 2-year DFS event. For reference, the four strategies “nomogram”, “radiomics score”, “location” and “tumour size” are displayed. The radiomics score-based nomogram model showed larger net benefit values than the other models (0.12-0.38).

According to the results of the multivariate analysis, we established a nomogram that incorporated the radiomics score and clinical factors for predicting DFS in patients with STS of the extremities and trunk treated with neoadjuvant radiotherapy. Each factor in the nomogram model was given a weighted point value (on a scale of 0–100 points) that implied survival prognosis (**Figure 3**). For instance, a high radiomics score was ascribed 0 points, which indicates a good prognosis. The lower the patient’s cumulative total score was, the lower the risk of a DFS event. The radiomics score-based nomogram model (Area Under Curve (AUC)=0.791) achieved significantly better predictive ability of 2-year DFS than the clinical stage (AUC=0.572, p=0.014), clinical T stage (AUC=0.518, p=0.008), radiomics score (AUC=0.658, p=0.049), tumour location (AUC=0.625, p=0.011) and tumour size (AUC=0.593, p=0.026) alone. Discrimination was evaluated by the C-index, and the radiomics score-based nomogram showed good discrimination, with a C-index of 0.781 (95% CI: 0.700–0.869, **Table 3**). The nomogram was validated internally with 1000 bootstrap resamples. The adjusted C-index was 0.738. With an AUC of 0.791 for 2-year DFS, we observed good discrimination and calibration in the radiomics score-based nomogram model (**Figures 4A, B**). DCA indicated that the use of the radiomics score-based nomogram model was associated with better benefit gains relative to the prediction of 2-year DFS compared to other models in the threshold probability range (0.12-0.38) (**Figure 4C**).

**Table 3:** C-index and AUC of radiomics score-based nomogram and clinical factors.

Variables	C-index	95%CI	Adjusted C-index (bootstrap resamples)	AUC for 2-year DFS
**Clinical stage**	0.608	0.501-0.716	0.589	0.572
**cT stage**	0.519	0.415-0.623	0.510	0.518
**Location**	0.591	0.496-0.686	0.569	0.625
**Tumour size**	0.616	0.510-0.723	0.623	0.593
**Radiomics score**	0.670	0.586-0.754	0.665	0.658
**Nomogram**	0.781	0.700-0.869	0.738	0.791

## Discussion

We developed a radiomics nomogram that combined the radiomics score with clinical factors and successfully predicted the DFS of patients with STS of the extremities and trunk treated with neoadjuvant radiotherapy. The radiomics-based nomogram exhibited discrimination performance with good reproducibility in the cohort. By using the radiomics nomogram model, patients were stratified into high-risk and low-risk groups according to the DFS outcome. The model that combined the radiomics score with clinical factors conferred better prognostic benefit than the model with clinical factors alone, with a significant difference in patient risk stratification.

With the current treatment pattern, neoadjuvant radiotherapy has important clinical value in the comprehensive treatment of STS (30). For preoperative RT, its advantages include the ability to treat with smaller RT fields and lower doses, both of which are associated with reduced permanent long-term toxicities. Other potential advantages of preoperative RT include the ability to render unresectable or marginally resectable tumours resectable, the potential to prevent tumour seeding of the operative bed or systemic circulation, and increased efficacy of RT from the good oxygenation of tissues (31, 32). Modern radiotherapy techniques may offer better patient outcomes with lower toxicities (33). For high-risk patients, intensive neoadjuvant therapy, including proton RT or a novel chemoradiotherapy regimen, may result in acceptable and manageable toxicity and favourable survival (34, 35). For patients with a good response to neoadjuvant therapy, RT can reduce the intensity of postoperative adjuvant therapy and even allow surgery to be avoided. However, how to screen patients and predict the neoadjuvant response is a hot topic. An imaging examination is the most effective non-invasive method. The rapid development of radiomics in recent years has provided more objective and accurate prediction indicators.

Due to its functional imaging capability, MRI is the preferred imaging modality for the diagnosis and staging of STS of the extremities and trunk (36, 37). Radiomics, a more high-throughput analysis method, can extract a large number of quantitative tumour imaging features. The features from radiologic images have the potential to maximize tumour characteristics that fail to be evaluated by visual inspection (38, 39). Several STSs radiomics relevant articles have been published, most of which focus on the pathological grading of the diagnosis or the differentiating between benign and malignant. For example, Yan et al. found that the STSs with low‐grade and high‐grade differentiation could be well distinguished by the radiomics nomogram (40). Wang et al. found that radiomics was accurate for distinguishing between malignant and benign soft-tissue masses (41). Due to the incidence and follow-up associated with soft tissue sarcomas, there are few studies focus on prognosis (**Supplementary Table 3**), and even fewer studies related to neoadjuvant radiotherapy. In the published studies, the neoadjuvant treatment models are mostly unclear or mixed, and most of them have not been separately analysed for the extremities and trunk STS. To our knowledge, this is the first study to combine pretreatment clinical factors and radiomics score for the development of a DFS prognostic nomogram model in STS of the extremities and trunk treated with neoadjuvant radiotherapy.

Previous studies have shown that several systems used to stage STS, such as clinical stage and pathological classification, are important prognostic stratification factors (7, 42), but the sensitivity of these models to predict prognosis still needs to be investigated further in the neoadjuvant radiotherapy setting. In our study, we extracted a total of 851 radiomics features from pretreatment T2FS imaging data. To select and reduce radiomics features, we used a robust statistical method based on LASSO-penalized Cox regression that has already provided good results according to the radiomics analyses of other cancers (43). Based on these results, we built a radiomics score-based nomogram model that showed significantly better performance for DFS prediction.

Our results show that the radiomics score-based nomogram could improve prognostic stratification according to tumour location and tumour size (the C-index increased). A future internal TRIPOD type 1B validation reflecting the prognostic stratification was not overfitted (44). The reason for this phenomenon may originate from the neoadjuvant treatment model. all the patients included in this study received neoadjuvant therapy, and this part of the population has the clinical characteristics of a large tumour burden and locally advanced stages. Although most patients are in similar clinical stages, potential heterogeneity (such as the sensitivity of treatment) exists among them. Therefore, the radiomics score model, which can capture a variety of information, was more sensitive than clinical features in prognostic prediction.

The present study has some limitations. First, we retrospectively analysed only patients who met the inclusion criteria; thus, the study may have selection bias. For example, patients who interrupted radiotherapy or completed radiotherapy and failed to undergo surgery due to the side effects and toxicity were not included in the analysis. These patients had a worse prognosis than those who received radiotherapy and surgery. Selective bias can also occur due to missing patient data in the retrospective study. Second, the sample size of the cohort was small. There was large heterogeneity between the two centres due to technical issues. Although there was technological heterogeneity between the two centres, in the consistency test, the consistency of the T2 model was reproducible and acceptable. Its impact on the prediction performances was within normal limits. Third, the histological subtypes of STS are heterogeneous (with more than 100 types), and a single retrospective study can hardly cover all the subtypes. One way to solve these problems may be to perform a prospective multicentre study with a larger sample size that is sufficiently large to cover most histological subtypes.

## Conclusions

We developed an MRI-based radiomics score from texture features that serves as an independent prognostic predictor for DFS in patients with STS of the extremities and trunk treated with neoadjuvant radiotherapy. The radiomics score-based nomogram could improve the prognostic stratification ability of traditional clinical factors, provide a more sensitive way to predict prognosis, and contribute to individualized therapy for STS patients.

## Data Availability Statement

The datasets presented in this article are not readily available because of the need to protect patient privacy and determine non-commercial use. Requests to access the datasets should be directed to corresponding authors.

## Author Contributions

SC, NL, and JJ contributed conception and design of the study. SC and YT organized the database. SC and NL performed the statistical analysis. All authors analysed and interpreted the detailing. SC and NL wrote the first draft of the manuscript. NL and JJ take final responsibility for this article. All authors contributed to the article and approved the submitted version.

## Funding

The present study was supported by National Natural Science Foundation of China (81871509); Fundamental Research Funds for Central Universities of the Central South University (3332019055); Capital’s Funds for Health Improvement and Research (2020-1-4021).

## Conflict of Interest

The authors declare that the research was conducted in the absence of any commercial or financial relationships that could be construed as a potential conflict of interest.

## Publisher’s Note

All claims expressed in this article are solely those of the authors and do not necessarily represent those of their affiliated organizations, or those of the publisher, the editors and the reviewers. Any product that may be evaluated in this article, or claim that may be made by its manufacturer, is not guaranteed or endorsed by the publisher.
