# Supplementary material for: Radiomics Analysis of Fat-Saturated T2-Weighted MRI Sequences for the Prediction of Prognosis in Soft Tissue Sarcoma of the Extremities and Trunk Treated With Neoadjuvant Radiotherapy
Source: Front Oncol. 2021 Sep 17;11:710649. doi: 10.3389/fonc.2021.710649 (PMC8484956; doi:10.3389/fonc.2021.710649)
Supplement: Supplementary file 1 [file DataSheet_1.docx]

Fig 1: Radiomics feature selection using LASSO regression mode

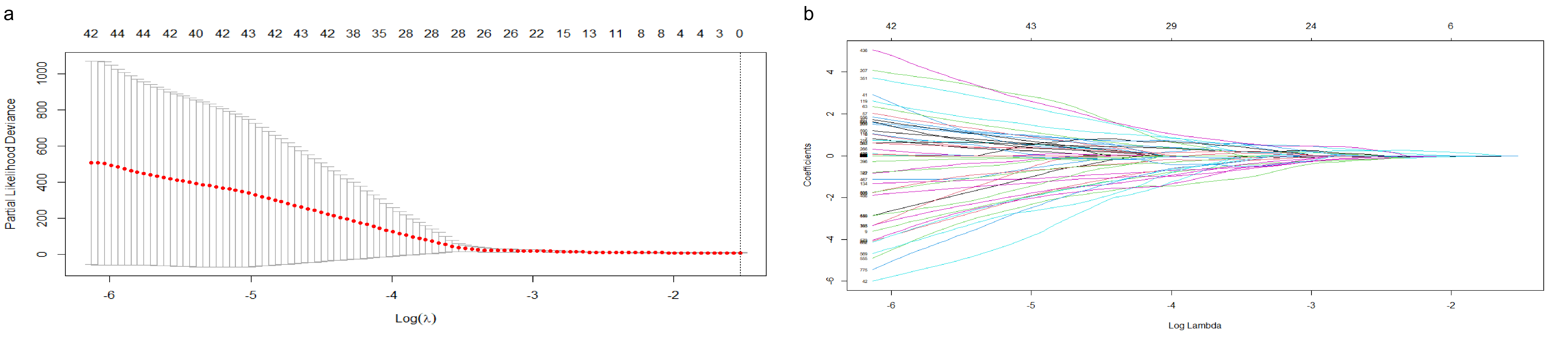


Fig 1a: Tuning parameter λ selection in the least absolute shrinkage and selection operator model used 10-fold cross-validation via minimum criteria. Area under the receiver operating characteristic curve was plotted versus the log λ. Dotted vertical lines were drawn at the optimal values by using the minimum criteria and the 1 standard error of the minimum criteria (the 1-SE criteria). The optimal λ value of 0.002175 with log (λ) =-2.663 was selected. Fig 1b: LASSO coefficient profiles of the 777 radiomics features. A coefficient profile plot was produced against the log(λ) sequence, and the optimal 42 non-zero coefficients were generated at the value selected using tenfold cross-validation.

Table 1: Radiomic Features coefficient of LASSO regression and Radiomic-score formula

| **Features class** | **Radiomic Features** | **Coefficient** |
| --- | --- | --- |
| First-order | Skewness | -0.83828214 |
|  | Minimum.1 | 2.01536448 |
|  | wavelet.HLL-Skewness | -1.33581432 |
|  | wavelet.HLL-Mean | -2.86438307 |
|  | wavelet.LHL-Skewness | 0.72702039 |
|  | wavelet.LHH-Kurtosis | -0.80086782 |
|  | wavelet.LLH-Skewness | -4.08300821 |
|  | wavelet.LLH-TotalEnergy | -0.27445512 |
|  | wavelet.HLH-Kurtosis | -1.89484426 |
|  | wavelet.HLH-Mean | 1.71803833 |
|  | wavelet.HHH-Skewness | -4.90602487 |
|  | wavelet.HHH-Median | 1.8488215 |
|  | wavelet.HHH-Kurtosis | -4.68376886 |
|  | wavelet.HHL-Median | 1.62766477 |
| GLCM | Imc2 | 2.91718786 |
|  | Imc1 | -5.99028015 |
|  | wavelet.HLL-InverseVariance | 2.61378106 |
|  | wavelet.LHL-Idn | 4.08948327 |
|  | wavelet.LHL-Correlation | 1.51248295 |
|  | wavelet.LLH-MCC | -3.33409139 |
|  | wavelet.LLH-Imc1 | 0.59720473 |
|  | wavelet.HLH-Imc1 | -1.12231708 |
| GLDM | wavelet.HLL-LargeDependenceHighGrayLevelEmphasis | 1.0466509 |
|  | wavelet.LHL-SmallDependenceLowGrayLevelEmphasis | -3.33589358 |
| GLRLM | ShortRunLowGrayLevelEmphasis | 2.35593093 |
|  | wavelet.HHH-RunVariance | -4.04251401 |
|  | wavelet.HHL-LowGrayLevelRunEmphasis | 0.07288406 |
|  | wavelet.HHL-RunVariance | -2.86971737 |
| GLSZM | wavelet.LHH-LowGrayLevelZoneEmphasis | 1.57953989 |
|  | wavelet.LHH-ZoneEntropy | 3.71609383 |
|  | wavelet.LLH-GrayLevelVariance | 0.10238227 |
|  | wavelet.HLH-SizeZoneNonUniformityNormalized | -1.77045852 |
|  | wavelet.HHH-LargeAreaEmphasis | 1.19324347 |
|  | wavelet.HHH-LargeAreaLowGrayLevelEmphasis | 0.5977806 |
|  | wavelet.HHH-SmallAreaEmphasis | -1.74984541 |
|  | wavelet.HHL-ZoneEntropy | -4.14246014 |
| NGTDM | wavelet.LHL-Strength | 0.32079438 |
|  | wavelet.LLH.1-Contrast | 5.05238111 |
|  | wavelet.LLL-Strength | -5.44518883 |
| Shape | SurfaceVolumeRatio | 0.80059592 |
|  | Maximum2DDiameterSlice | 1.03183688 |
|  | Flatness | -3.62143697 |

NOTE. Abbreviations: first-order, first-order statistics; GLCM, grey-level cooccurrence matrix (GLCM); GLDM, grey-level dependence matrix; GLRLM, grey-level run length matrix; GLSZM, grey-level size zone matrix; NGTDM, grey tone difference matrix.

Radiomic-score formula:

Radiomic-score= -0.83828214× (Skewness) + 2.01536448× (Minimum.1) -1.33581432× (wavelet.HLL-Skewness) -2.86438307× (wavelet.HLL-Mean)+ 0.72702039× (wavelet.LHL-Skewness) -0.80086782× (wavelet.LHH-Kurtosis) -4.08300821×( wavelet.LLH-Skewness) -0.27445512 ×( wavelet.LLH-TotalEnergy) -1.89484426×(wavelet.HLH-Kurtosis)+ 1.71803833×(wavelet.HLH-Mean)-4.90602487×(wavelet.HHH-Skewness)+ 1.8488215×(wavelet.HHH-Median)-4.68376886×(wavelet.HHH-Kurtosis)+ 1.62766477×(wavelet.HHL-Median)+ 2.91718786 ×(Imc2) -5.99028015×(Imc1)+ 2.61378106×(wavelet.HLL-InverseVariance)+ 4.08948327×(wavelet.LHL-Idn)+ 1.51248295×(wavelet.LHL-Correlation)-3.33409139×(wavelet.LLH-MCC)+ 0.59720473×(wavelet.LLH-Imc1) -1.12231708×(wavelet.HLH-Imc1)+ 1.0466509×(wavelet.HLL-LargeDependenceHighGrayLevelEmphasis) -3.33589358 ×(wavelet.LHL-SmallDependenceLowGrayLevelEmphasis)+ 2.35593093×(ShortRunLowGrayLevelEmphasis) -4.04251401×(wavelet.HHH-RunVariance)+ 0.07288406×(wavelet.HHL-LowGrayLevelRunEmphasis) -2.86971737×(wavelet.HHL-RunVariance)+ 1.57953989×(wavelet.LHH-LowGrayLevelZoneEmphasis)+ 3.71609383×(wavelet.LHH-ZoneEntropy)+ 0.10238227×(wavelet.LLH-GrayLevelVariance) -1.77045852×(wavelet.HLH-SizeZoneNonUniformityNormalized)+ 1.19324347×(wavelet.HHH-LargeAreaEmphasis)+ 0.5977806×(wavelet.HHH-LargeAreaLowGrayLevelEmphasis) -1.74984541×(wavelet.HHH-SmallAreaEmphasis)-4.14246014 ×(wavelet.HHL-ZoneEntropy) +0.32079438×(wavelet.LHL-Strength)+ 5.05238111×(wavelet.LLH.1-Contrast) -5.44518883×(wavelet.LLL-Strength)+ 0.80059592×(SurfaceVolumeRatio)+ 1.03183688×(Maximum2DDiameterSlice) -3.62143697×(Flatness)

**Table 2: Patient characteristics**

| **Characteristics** | NCC (**n =20**) | TCIA（n=42） | | *P* |
| --- | --- | --- | --- | --- |
| Gender |  |  | |  |
| Male | 13(65.0) | 19(45.2) | 0.146 | |
| Female | 7(35.0) | 23(54.8) |  | |
| Age at diagnosis (y), mean±SD | 47.8±20.1 | 55.7±16.7 | 0.100 | |
| Histotype |  |  |  | |
| Undifferentiated sarcoma | 1(5.0) | 13(31.0) | 0.020 | |
| liposarcoma | 5(25.0) | 10(23.8) |  | |
| Synovial sarcoma | 2(10.0) | 3(7.1) |  | |
| Leiomyosarcoma | 0 | 10(23.8) |  | |
| Fibrosarcoma | 3(15.0) | 1(2.4) |  | |
| Other^a^ | 9(45.0) | 5(11.9) |  | |
| Grade |  |  |  | |
| Low | 3(15.0) | 4(9.5) | 0.462 | |
| Intermediate | 3(15.0) | 13(31.0) |  | |
| High | 10(50.0) | 22(52.4) |  | |
| Unkown | 4(20.0) | 3(7.1) |  | |
| Location |  |  |  | |
| Trunk | 1(5.0) | 8(19.0) | 0.142 | |
| Extremities | 19(95.0) | 34(81.0) |  | |
| MRI T stage |  |  |  | |
| cT1 | 3(15.0) | 4(9.5) | 0.524 | |
| cT2 | 17(85.0) | 38(90.5) |  | |
| MRI N stage |  |  |  | |
| cN0 | 20(100.0) | 42(100%) | - | |
| Depth |  |  |  | |
| Superficial | 6(30.0) | 10(23.8) | 0.603 | |
| Deep | 14(70.0) | 32(76.2) |  | |
| Clinical stage |  |  |  | |
| I | 3(15.0) | 4(9.5) | 0.689 | |
| II | 9(45.0) | 18(42.9) |  | |
| III | 8(40.0) | 20(47.6) |  | |
| Treatment |  |  |  | |
| Radiotherapy + Surgery | 20(100.0) | 29(69.0) | 0.005 | |
| Radiotherapy + Surgery + Chemotherapy | 0 | 13(31.0) |  | |

NOTE. Data are reported as No. (%) Abbreviations: NCC, our institute; TCIA, The Cancer Imaging Archive.

a. Including epithelioid sarcoma, myxofibrosarcoma, extraskeletal high grade osteogenic sarcoma, etc.

**Table 3. Summary of MRI-radiomics Study for prognosis in soft tissue sarcoma**

| **Author** | **Year** | **Enrolled interval** | **Study design** | **Sample size**  **(n)** | **Number of cases of extremities and trunk**  **(n)** | **Number of cases of neoadjuvant radiotherapy**  **(n)** | **Outcome** |
| --- | --- | --- | --- | --- | --- | --- | --- |
| Vallières (1) | 2015 | 2004-2011 | Retrospective | 51 | 51 | 44 | Relapse |
| Vallières (2) | 2017 | 2004-2011 | Retrospective | 30 | 30 | 26 | Relapse |
| Meyer (3) | 2019 | unknow | Retrospective | 29 | unknow | unknow | Long-term survival |
| Spraker (4) | 2019 | 2000-2017 | Retrospective | 226 | unknow | 226 | Long-term survival |
| Crombé (5) | 2019 | 2017-2018 | Prospective | 25 | 25 | unknow | Therapy response |
| Crombé (6) | 2019 | 2007-2017 | Retrospective | 65 | 65 | 0 | Therapy response |
| Tian (7) | 2020 | 2009-2018 | Retrospective | 77 | unknow | unknow | Relapse |
| Crombé (8) | 2020 | 2006-2016 | Retrospective | 70 | 70 | 0 | Relapse, Long-term survival |
| Crombé (9) | 2020 | 2008-2017 | Retrospective | 35 | 35 | 0 | Relapse, Long-term survival |
| Crombé (10) | 2020 | 2012-2018 | Retrospective | 50 | 50 | 0 | Relapse, Long-term survival |
| Gao (11) | 2020 | unknow | Prospective | 30 | 30 | 30 | Therapy response |
| Crombé (12) | 2020 | unknow | Retrospective | 42 | 34 | 4 | Relapse, Long-term survival |
| Crombé(13) | 2020 | 2008-2018 | Retrospective | 21 | 21 | 0 | Relapse, Long-term survival |

NOTE. Relapse: Includes patients with local recurrence or distant metastases.

1. Vallières M, Freeman CR, Skamene SR, El Naqa I. A radiomics model from joint FDG-PET and MRI texture features for the prediction of lung metastases in soft-tissue sarcomas of the extremities. *Phys Med Biol* (2015) 60(14):5471-96. Epub 2015/06/30. doi: 10.1088/0031-9155/60/14/5471. PubMed PMID: 26119045.

2. Vallières M, Laberge S, Diamant A, El Naqa I. Enhancement of multimodality texture-based prediction models via optimization of PET and MR image acquisition protocols: a proof of concept. *Phys Med Biol* (2017) 62(22):8536-65. Epub 2017/09/06. doi: 10.1088/1361-6560/aa8a49. PubMed PMID: 28872054.

3. Meyer HJ, Renatus K, Höhn AK, Hamerla G, Schopow N, Fakler J, et al. Texture analysis parameters derived from T1-and T2-weighted magnetic resonance images can reflect Ki67 index in soft tissue sarcoma. *Surg Oncol* (2019) 30:92-7. Epub 2019/09/11. doi: 10.1016/j.suronc.2019.06.006. PubMed PMID: 31500794.

4. Spraker MB, Wootton LS, Hippe DS, Ball KC, Peeken JC, Macomber MW, et al. MRI Radiomic Features Are Independently Associated With Overall Survival in Soft Tissue Sarcoma. *Adv Radiat Oncol* (2019) 4(2):413-21. Epub 2019/04/24. doi: 10.1016/j.adro.2019.02.003. PubMed PMID: 31011687; PubMed Central PMCID: PMCPMC6460235.

5. Crombé A, Saut O, Guigui J, Italiano A, Buy X, Kind M. Influence of temporal parameters of DCE-MRI on the quantification of heterogeneity in tumor vascularization. *J Magn Reson Imaging* (2019) 50(6):1773-88. Epub 2019/04/14. doi: 10.1002/jmri.26753. PubMed PMID: 30980697.

6. Crombé A, Périer C, Kind M, De Senneville BD, Le Loarer F, Italiano A, et al. T(2) -based MRI Delta-radiomics improve response prediction in soft-tissue sarcomas treated by neoadjuvant chemotherapy. *J Magn Reson Imaging* (2019) 50(2):497-510. Epub 2018/12/21. doi: 10.1002/jmri.26589. PubMed PMID: 30569552.

7. Tian L, Zhang D, Bao S, Nie P, Hao D, Liu Y, et al. Radiomics-based machine-learning method for prediction of distant metastasis from soft-tissue sarcomas. *Clin Radiol* (2021) 76(2):158.e19-.e25. Epub 2020/12/10. doi: 10.1016/j.crad.2020.08.038. PubMed PMID: 33293024.

8. Crombé A, Kind M, Fadli D, Le Loarer F, Italiano A, Buy X, et al. Intensity harmonization techniques influence radiomics features and radiomics-based predictions in sarcoma patients. *Sci Rep* (2020) 10(1):15496. Epub 2020/09/25. doi: 10.1038/s41598-020-72535-0. PubMed PMID: 32968131; PubMed Central PMCID: PMCPMC7511974.

9. Crombé A, Le Loarer F, Sitbon M, Italiano A, Stoeckle E, Buy X, et al. Can radiomics improve the prediction of metastatic relapse of myxoid/round cell liposarcomas? *Eur Radiol* (2020) 30(5):2413-24. Epub 2020/01/19. doi: 10.1007/s00330-019-06562-5. PubMed PMID: 31953663.

10. Crombé A, Fadli D, Buy X, Italiano A, Saut O, Kind M. High-Grade Soft-Tissue Sarcomas: Can Optimizing Dynamic Contrast-Enhanced MRI Postprocessing Improve Prognostic Radiomics Models? *J Magn Reson Imaging* (2020) 52(1):282-97. Epub 2020/01/11. doi: 10.1002/jmri.27040. PubMed PMID: 31922323.

11. Gao Y, Kalbasi A, Hsu W, Ruan D, Fu J, Shao J, et al. Treatment effect prediction for sarcoma patients treated with preoperative radiotherapy using radiomics features from longitudinal diffusion-weighted MRIs. *Phys Med Biol* (2020) 65(17):175006. Epub 2020/06/20. doi: 10.1088/1361-6560/ab9e58. PubMed PMID: 32554891.

12. Crombé A, Kind M, Ray-Coquard I, Isambert N, Chevreau C, André T, et al. Progressive Desmoid Tumor: Radiomics Compared With Conventional Response Criteria for Predicting Progression During Systemic Therapy-A Multicenter Study by the French Sarcoma Group. *AJR Am J Roentgenol* (2020) 215(6):1539-48. Epub 2020/09/30. doi: 10.2214/ajr.19.22635. PubMed PMID: 32991215.

13. Crombe A, Sitbon M, Stoeckle E, Italiano A, Buy X, Le Loarer F, et al. Magnetic resonance imaging assessment of chemotherapy-related adipocytic maturation in myxoid/round cell liposarcomas: specificity and prognostic value. *Br J Radiol* (2020) 93(1110):20190794. Epub 2020/02/28. doi: 10.1259/bjr.20190794. PubMed PMID: 32105502.

**References:**
